# Dissecting the mechanisms involved in anti-human T-lymphocyte immunoglobulin (ATG)-induced tolerance in the setting of allogeneic stem cell transplantation - potential implications for graft versus host disease

**DOI:** 10.18632/oncotarget.21797

**Published:** 2017-10-11

**Authors:** Katia Beider, David Naor, Valeria Voevoda, Olga Ostrovsky, Hanna Bitner, Evgenia Rosenberg, Nira Varda-Bloom, Victoria Marcu-Malina, Jonathan Canaani, Ivetta Danilesko, Avichai Shimoni, Arnon Nagler

**Affiliations:** ^1^ Hematology Division, Chaim Sheba Medical Center and Tel Aviv University, Tel-Hashomer, Ramat Gan, Israel; 2 Lautenberg Center for Immunology and Cancer Research, Faculty of Medicine, Hebrew University of Jerusalem, Jerusalem, Israel

**Keywords:** ATG, Treg, TGFβ, Immunology and Microbiology Section, Immune response, Immunity

## Abstract

Polyclonal anti-human thymocyte globulins (ATG) have been recently shown to significantly reduce the incidence of graft versus host disease (GVHD) post allogeneic stem cell transplantation (HSCT) from both sibling and unrelated donors. Induction of regulatory T cells has been suggested as one of the possible mechanisms. The aim of current study was to further characterize the T cell populations induced by ATG treatment and to delineate the mechanisms involved in ATG-induced tolerance. Phenotypic characterization revealed a significant increase in the expression of FoxP3, GITR, CD95, PD-1 and ICOS as well as the complement inhibitory molecules CD55, CD58 and CD59 on CD4+CD25+ T cells upon ATG treatment. Addition of ATG-treated cells to autologous and allogeneic peripheral blood mononuclear cells (PBMCs) stimulated with anti-CD3/anti-CD28 antibodies resulted in significant inhibition of proliferation. Moreover, T-cell activation and IFNγ secretion were reduced in the presence of ATG-induced Treg cells. The CD4+CD25+CD127-low Treg fraction sorted from ATG-treated culture demonstrated greater suppressive potency than negative fraction. Conditioned medium produced by ATG-treated but not IgG-treated cells contained TGFβ and suppressed T cell proliferation and activation in a TGFβ receptor-dependent manner. TGFβ receptor kinase inhibitor SB431542 interfered with the suppressive activity of ATG-primed cells, enabling partial rescue of proliferation and IFNγ secretion. Moreover, SB431542 prevented Treg phenotype induction upon ATG treatment. Altogether, our data reveal the role of TGFβ signaling in ATG-mediated immunosuppression and further support the use of ATG, a potent inducer of regulatory T cells, for prevention of GVHD post HSCT and potentially other therapeutic applications.

## INTRODUCTION

Allogeneic hematopoietic stem cell transplantation (HSCT) is an established treatment for many hematological neoplasms including leukemia, lymphoma, and myeloma [1-5]. T cells and natural killer (NK) cells within the graft make the predominant contribution to graft-*versus*-tumor (GVT) activity [3, 6]. However, alloreactive donor immune cells are also responsible for one of the main reasons for transplant-related morbidity and mortality, namely graft-*versus*-host disease (GVHD) [7, 8]. In GVHD, recipient tissues are attacked by alloreactive donor non-regulatory effector CD4+ and CD8+ T cells [9, 10]. Depletion of T cells from the allogeneic graft significantly reduces the incidence of GVHD, albeit at the price of delayed immune reconstitution [11], poorer donor-cell engraftment, as well as reduced GVT effect [12]. Accordingly, the goal of ongoing research is to preserve donor T cells in the graft, while concomitantly attempting to reduce donor T-cell-mediated acute and chronic GVHD [13]. CD4+CD25+FoxP3+ regulatory T cells (Tregs) comprise 5% to 10% of circulating CD4+ T cells. They are essential in maintaining peripheral self-tolerance and preventing inflammatory disease [14-16]. Several studies have suggested that Tregs also play a central role in the establishment and maintenance of immune tolerance following HSCT. Indeed, depletion of CD25+ cells from splenocytes in a murine model resulted in increased GVHD severity and lethality after major histocompatibility complex-mismatched bone marrow transplantation, providing evidence that Tregs ameliorate the detrimental effects of alloreactive donor effector T cells in the host [16, 17]. Similarly, Treg transplantation at high ratios could completely protect recipient mice from GVHD, even when lethal doses of effector T cells were administered [17, 18]. Notably, clinical studies have demonstrated reduced CD4+FoxP3+ T-cell frequencies in the blood and in the mucosal tissues of patients with GVHD [19]. Recently, adoptive transfer of freshly isolated or *in vitro*-expanded Tregs from peripheral blood or cord blood was used in early clinical trials facilitating immune reconstitution with no increased risk of GVHD [20-23]. Rabbit anti-thymocyte globulins (ATG- Fresenius) (Neovii-Biotech, Graefelfing, Germany) are a set of polyclonal antibodies (IgG fraction) directed against a variety of immune cell epitopes, purified from the serum of rabbits that were immunized with human T cells [24]. ATG is administered as part of the transplantation conditioning regimen to reduce the incidence and severity of GVHD by *in vivo* T-cell depletion. Due to their long half-life in human plasma (up to 6 weeks), ATG can persist in the blood for several weeks after infusion [25, 26] and induce apoptosis of donor T cells passively transferred with the graft. The beneficial effects of pre-transplant ATG for GVHD prevention have been demonstrated in several clinical studies [27-31]. Recently it was shown that pre-transplant ATG selectively depletes donor naive T cells and central memory CD4+ T cells, while it relatively preserves other T cell subsets. Specifically, Treg were not affected by pre-transplant ATG [32]. Since Treg cells can mediate immune tolerance [33, 34], their persistence might have also prevented GVHD.

The ability of ATG to promote Treg phenotype acquisition *ex vivo* has been demonstrated in previous studies. Thus, *in vitro* treatment with Thymoglobulin (rabbit anti-human ATG produced by immunization against thymocytes, Genzyme) efficiently induced the expression of Treg markers and provided suppressive activity to generated Treg cells [35-37]. Furthermore, our previous work demonstrated that ATG-F (produced by rabbit immunization against the human T lymphoblastoid cell line Jurkat, Neovii Biotech) promoted Treg cell generation *in vitro*, and suggested that ATG induces expansion of regulatory T cells in patients undergoing allogeneic HSCT [38]. However, there are controversial data with regard to the potential of ATG to promote expression of regulatory phenotype in T cells. Thus, Broady et al. demonstrated transient up-regulation of Foxp3 expression upon ATG treatment without the acquisition of suppressive potential that was related to activated rather than suppressive phenotype [39]. Therefore, it is important to re-evaluate the effects of ATG in regulatory phenotype induction and to determine the mechanisms underlying this activity. Our work demonstrates that *in vitro* treatment with ATG is capable of inducing functional Treg cells. The suppressive ability of ATG-induced cells is both contact and soluble-factors dependent and is partially promoted by TGFβ signaling. Altogether, our data further support the use of ATG-F, a potent inducer of Treg cells, for prevention of GVHD post HSCT and potentially for other therapeutic applications.

## RESULTS

### ATG induces Treg phenotype acquisition in CD4+ T cells

**Figure 1:**
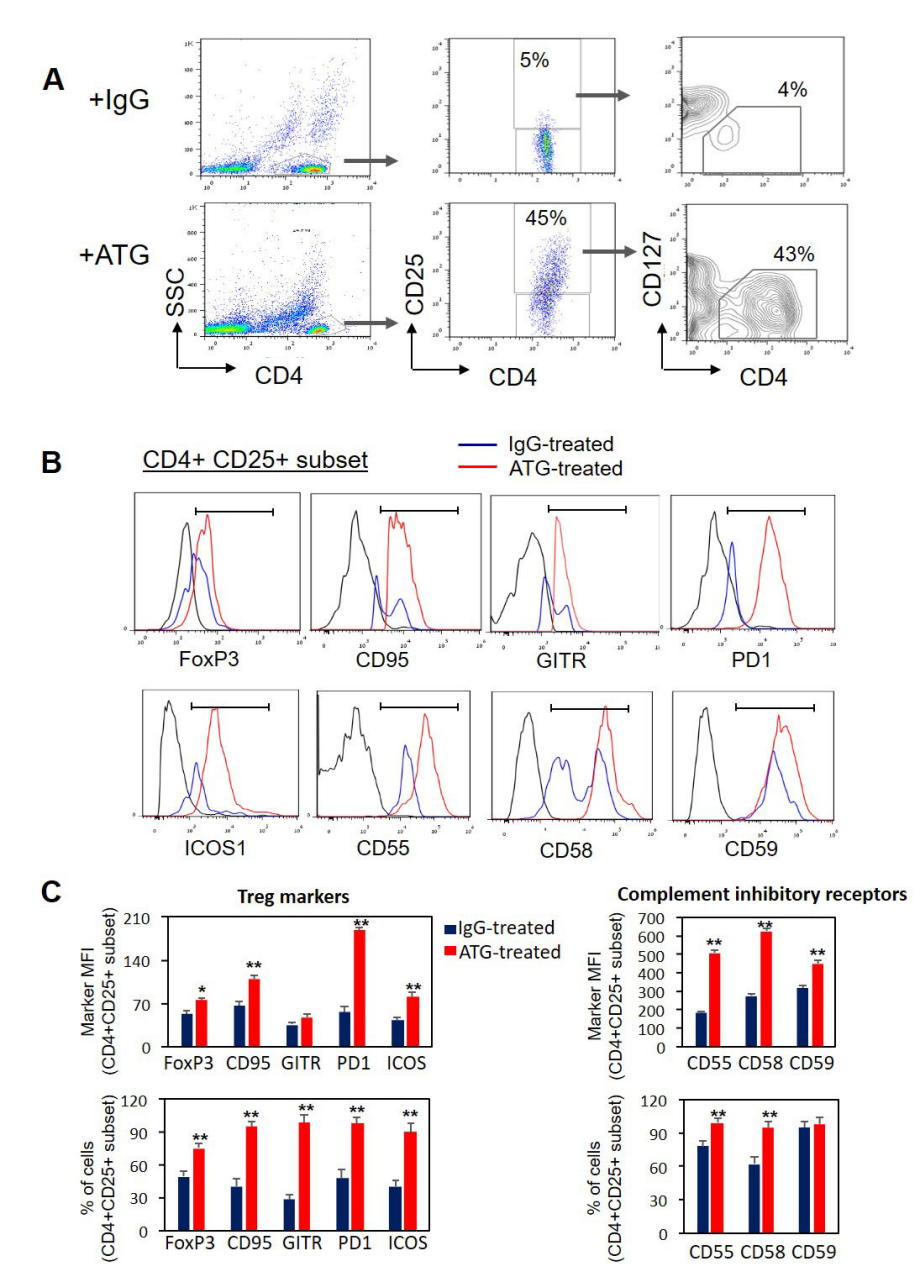
ATG treatment induces the expression of Treg markers and complement inhibitory receptors Normal PBMCs were incubated during 48 hours with ATG (60 µg/ml) or rabbit IgG. Cell surface staining for CD4, CD25, CD127, CD95, GITR, ICOS, PD-1, CD55, CD58, CD59 and intra-cellular staining for FoxP3 was performed. **A.** Representative plots showing CD25 and CD127 expression on gated CD4+ cells. CD4+CD25+CD127-low subset of Treg cells is enumerated. **B.** Representative histograms showing the expression of Treg markers and complement inhibitory receptors in the CD4+CD25+ cell subset. **C.** Mean fluorescence intensity (MFI) and percentage of CD4+ CD25+ cells expressing the specific markers are presented as mean of triplicates (**p < 0.01). Data are representative of four independent experiments.

First, to assess the effect of *in vitro* ATG treatment on T cell phenotype, freshly purified PBMCs from healthy donors were exposed during 48 hours to ATG (60 µg/ml) (Neovii-Biotech, Graefelfing, Germany) or to rabbit IgG as a control. Pharmacokinetics studies [40] suggest that chosen *in vitro* ATG concentration (60 µg/ml) is achievable in patients administered with 30 mg/kg [31] or 60 mg/kg ATG-F [28]. Markers associated with Treg phenotype were evaluated by flow cytometry. As shown in Figure 1A, ATG treatment induced marked increase in the frequency of CD4+CD25+CD127-low Treg population in culture. Moreover, expression of Treg markers FoxP3, CD95, GITR, PD-1 and ICOS was significantly increased on the gated CD4+CD25+ cells following the treatment with ATG compared with IgG treatment (Figure 1B, 1C). In addition, ATG treatment up-regulated the expression of complement inhibitory receptors CD55, CD58 and CD59 on the surface of CD4+CD25+ cell subset. These findings were consistent in all samples from different donors (*n* = 4) that were analyzed and indicated the acquisition of Treg phenotype in CD4+ T cells upon exposure to ATG *in vitro*. The increase in the expression of complement inhibitory receptors following ATG treatment suggests the reduced sensitivity of generated Treg cells to complement-dependent cytotoxicity.

**Figure 2:**
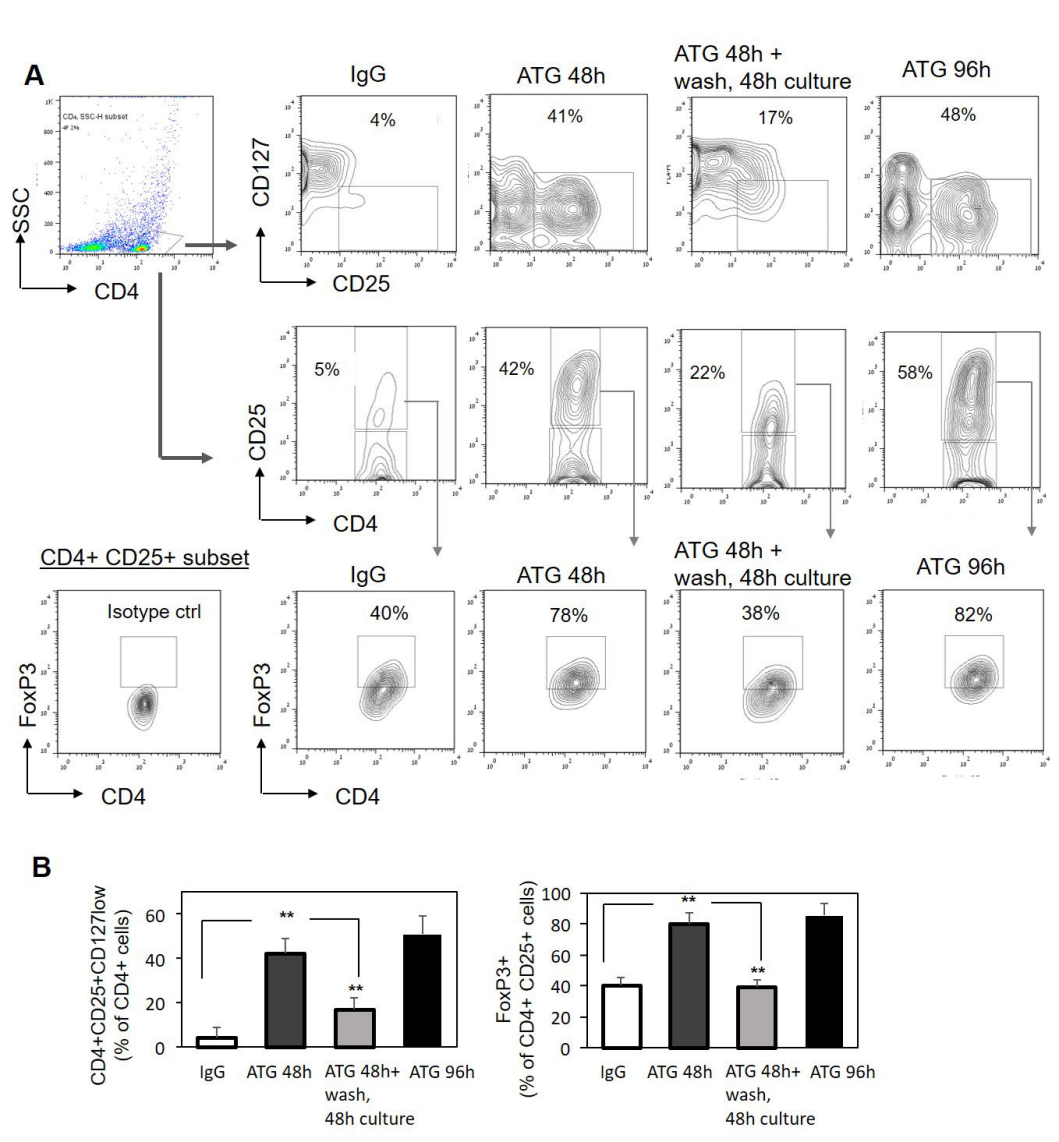
ATG-mediated induction of Treg phenotype is reversible PBMCs were incubated for 48 hours or 96 hours with ATG (60 µg/ml) or rabbit IgG. Alternatively, PBMCs were exposed to ATG for 48 hours, then ATG was removed, cells were washed with PBSx1 three times and re-plated in fresh medium without ATG for additional 48 hours. Cells were analyzed by flow cytometry for CD4, CD25, CD127 and FoxP3 expression. **A.** Representative plots demonstrating the frequency of CD4+CD25+CD127low and CD4+CD25+FoxP3+ Treg cells following the treatments. **B.** Percentage of CD4-positive cells co-expressing high CD25 and low CD127 (left panel), or high CD25 and high FoxP3 (right panel) presented as mean of triplicates ±STDEV (**p < 0.01).

To evaluate the stability of the acquired Treg phenotype, PBMCs were exposed to ATG for 48 hours, then ATG was removed and the cells were washed and re-plated for an additional 48 hours. As shown in Figure 2, ATG removal resulted in a subsequent decrease in Treg markers expression, including CD25 and FoxP3 down-regulation and up-regulation of CD127. This reversion was not simply related to the prolonged culture time, since the cells incubated for the same period of 96 hours with the continuous exposure to ATG demonstrated stable Treg phenotype (Figure 2A, 2B). Therefore, we can conclude that ATG-mediated Treg induction is a reversible phenomenon and the presence of ATG is necessary to promote and preserve this effect. However, taking into account the long half-life time of ATG in the serum (elimination half-life of 30 days [41]) one can suggest that ATG persistence before and during the first weeks after allogeneic HSCT will enable Treg generation which may decline as ATG serum levels decrease.

### ATG-induced Treg cells suppress autologous T cell proliferation

**Figure 3:**
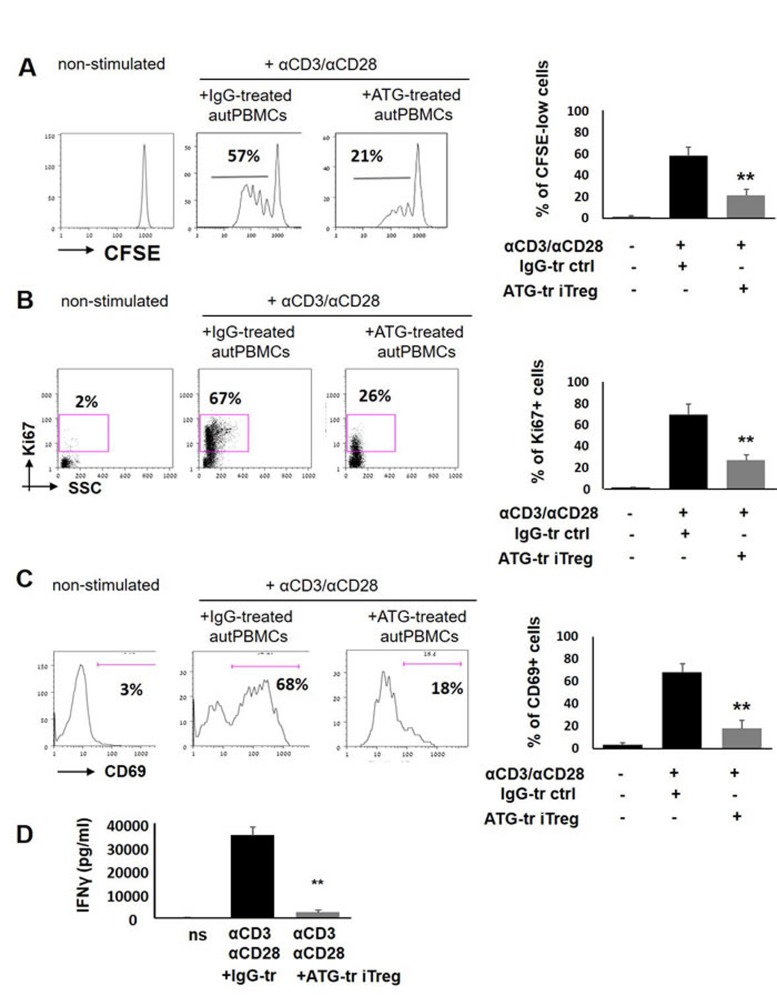
ATG-induced Treg cells suppress T cell proliferation and activation PBMCs were stimulated with anti-CD3/anti-CD28 antibodies, in the presence of autologous ATG- or rabbit IgG-pretreated cells (at 1:1 ratio), for 5 days of incubation. Suppressive effect of ATG-induced Treg-enriched cells on proliferation was measured by CFSE dilution method **A.** or by Ki67 expression **B.** Percentage of proliferated CD3+ T cells is shown as mean of triplicates ±STDEV (**p < 0.01). Data are representative of three independent experiments. **C.** Percentage of CD3-positive cells expressing the activation marker CD69 at day 5 of stimulation, presented as mean of triplicates ±STDEV (**p < 0.01). Left panes of A, B and C shows representative staining plots, while right panels show the percentage of proliferated or activated cells. **D**. IFNγ levels at day 5 of stimulation in the conditioned medium were determined by ELISA, presented as mean of triplicates ±STDEV (**p < 0.01). Tr = treated.

Next, the functional properties of ATG-induced Treg (iTreg) cells were tested. To evaluate the suppressive potential of induced Treg cells, ATG-treated cells were applied to the proliferation assay of autologous PBMCs stimulated with anti-CD3/anti-CD28 soluble antibodies. A proliferative response of gated CD3-positive cells was measured using CFSE dye dilution method and by testing Ki67 marker expression. As demonstrated in Figure 3, addition of ATG-treated iTreg cells but not of IgG-treated control cells resulted in significant suppression of autologous T cell proliferation, decreasing the percent of CFSE-low and Ki67-positive cells, respectively (Figure 3A, 3B). Accordingly, ATG-treated iTreg cells but not IgG-treated control cells were capable of reducing the expression of T cell activation marker CD69 by 50-60% upon CD3/CD28 stimulation (Figure 3C). Furthermore, the secretion of the pro-inflammatory cytokine IFNγ in response to CD3/CD28 stimulation was nearly fully abrogated in the presence of ATG-treated iTreg cells (Figure 3D). These findings indicate that ATG-induced Treg cells are functionally competent and capable of suppressing the proliferation and activation of autologous T cells.

### CD8+ effector T cells are the most sensitive to ATG-induced Treg cell-mediated suppression

**Figure 4:**
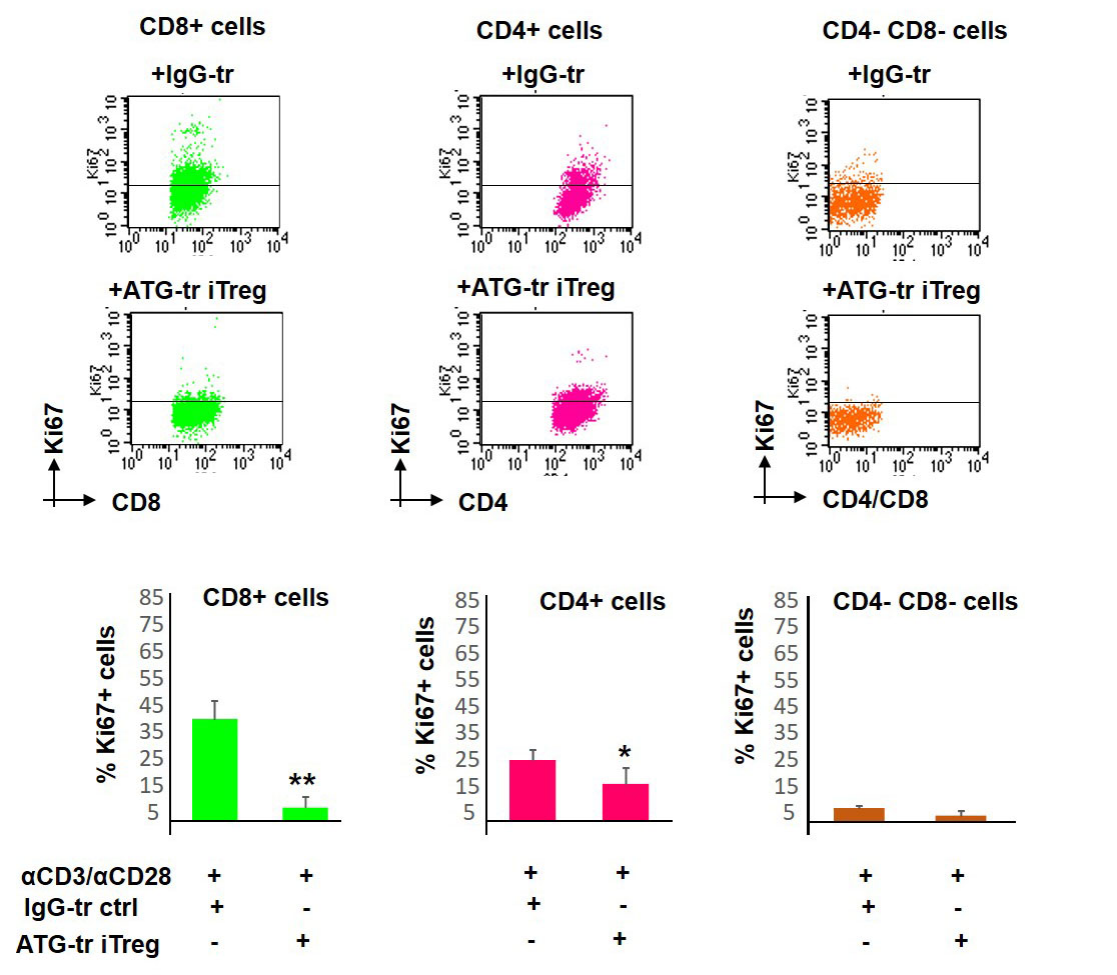
Proliferative response of different T cell subsets PBMCs were stimulated with anti-CD3/anti-CD28 antibodies, in the presence of autologous ATG- or rabbit IgG-pretreated cells (at 1:1 ratio), for 5 days of incubation. Proliferation of CD8-positive, CD4-positvie or CD4/CD8-negativie cells was determined by Ki67 expression. Representative plots showing Ki67 expression in specific cell sub-populations. Suppressive potential of ATG-induced Treg cells on different sub-populations, percentage of proliferated cells is presented as mean of triplicates ±STDEV at the lower panels (*p < 0.05, **p < 0.01). Data are representative of three independent experiments. Tr = treated.

Next, we wanted to delineate the response of specific T cell subsets to ATG-iTreg-mediated suppression. Therefore, the differential response of CD3+CD4+ T helper and CD3+CD8+ T effector cells to iTreg-mediated suppression of proliferation was evaluated. Interestingly, the most profound suppressive effect was detected in CD8+ effector population, reducing the percent of Ki67-positive proliferating cells from 47% (±7%) in control group to 8% (±3%), thus resulting in an 83% proliferation reduction (**p < 0.01) following the co-culture with ATG iTreg cells. In comparison to CD8+ effector cells, CD4+ T helper cells demonstrated lower initial proliferative rates in response to CD3/CD28 activation (25% ±3% in control group) and were less responsive to iTreg-mediated suppression, showing 15% ±5% proliferation, and thus 40% suppression (*p < 0.05). The remaining CD4-negative CD8-negative non-T cells were barely activated by CD3/CD28 stimulation and were not affected by the iTreg cell (Figure 4).

### ATG-induced Treg cells are able to suppress both autologous and allogeneic cell proliferation

**Figure 5:**
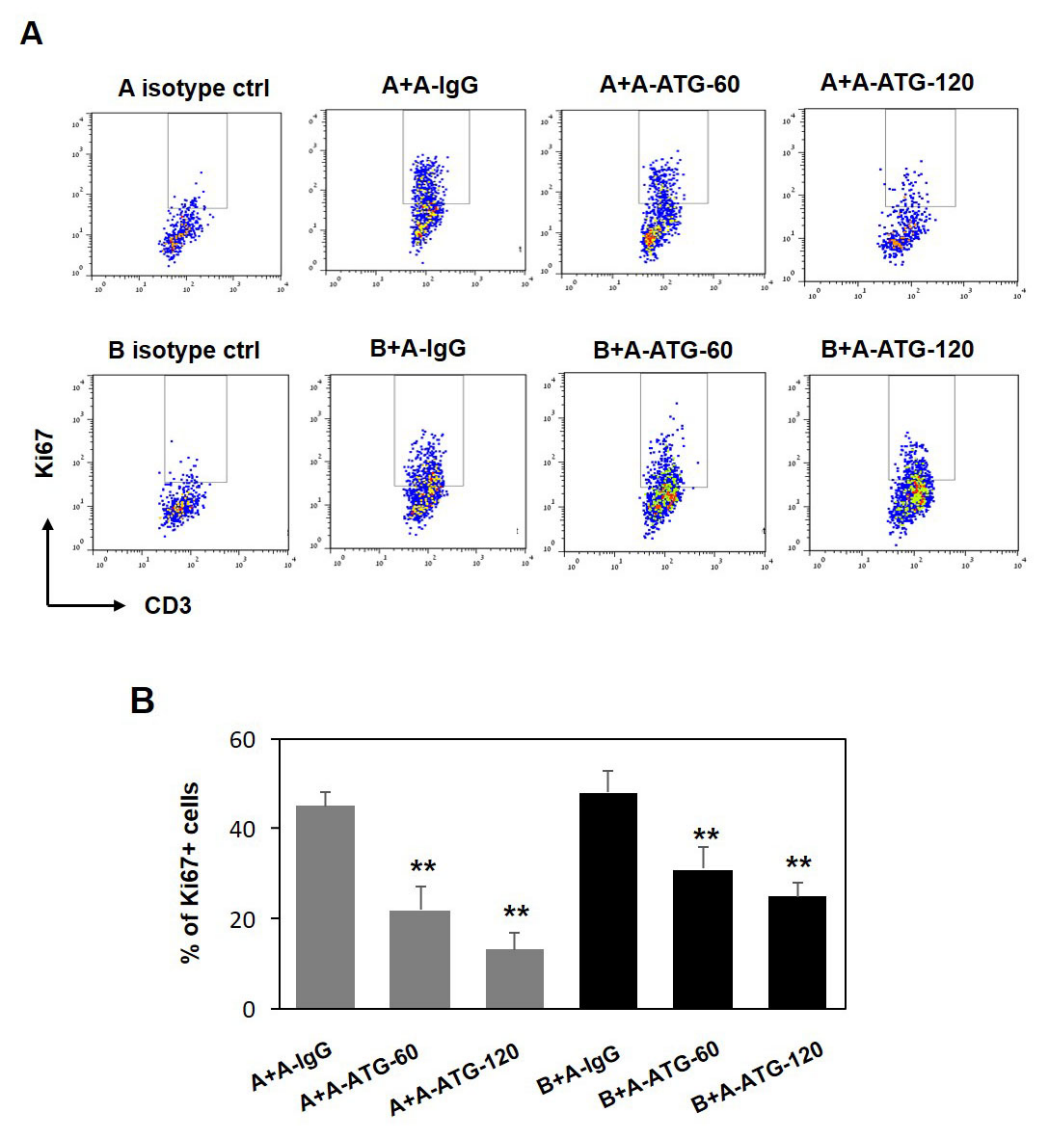
ATG-induced Treg cells are capable to supress both autologus and allogeneic T cell proliferation PBMCs from donor A were incubated during 48 hours with either IgG or ATG (60 or 120 µg/ml), and applied to autologous PBMCs (donor A), or to allogeneic PBMCs (Donor B) (at ratio 1:1), stimulated with anti-CD3/anti-CD28 for 5 days. Proliferation was measured by Ki67 expression. **A.** Representative plots demonstrating Ki67 expression in CD3-poisitve cells. **B.** Percentage of proliferated CD3+ T cells is presented by the bars as mean of triplicates ±STDEV (**p < 0.01). Data are representative of two independent experiments.

Our next goal was to evaluate whether the suppressive effect of ATG-induced Treg cells is restricted to autologous cells or whether it could be extended to allogeneic T cells as well. Indeed, in addition to autologous cells, we observed a potent suppressive effect mediated by ATG-treated Treg cells against allogeneic effector T cells stimulated with anti-CD3/anti-CD28 antibodies that was comparable with inhibition of autologous T cells (Figure 5).

### Sorting of tolerizing population from ATG-induced cells

**Figure 6:**
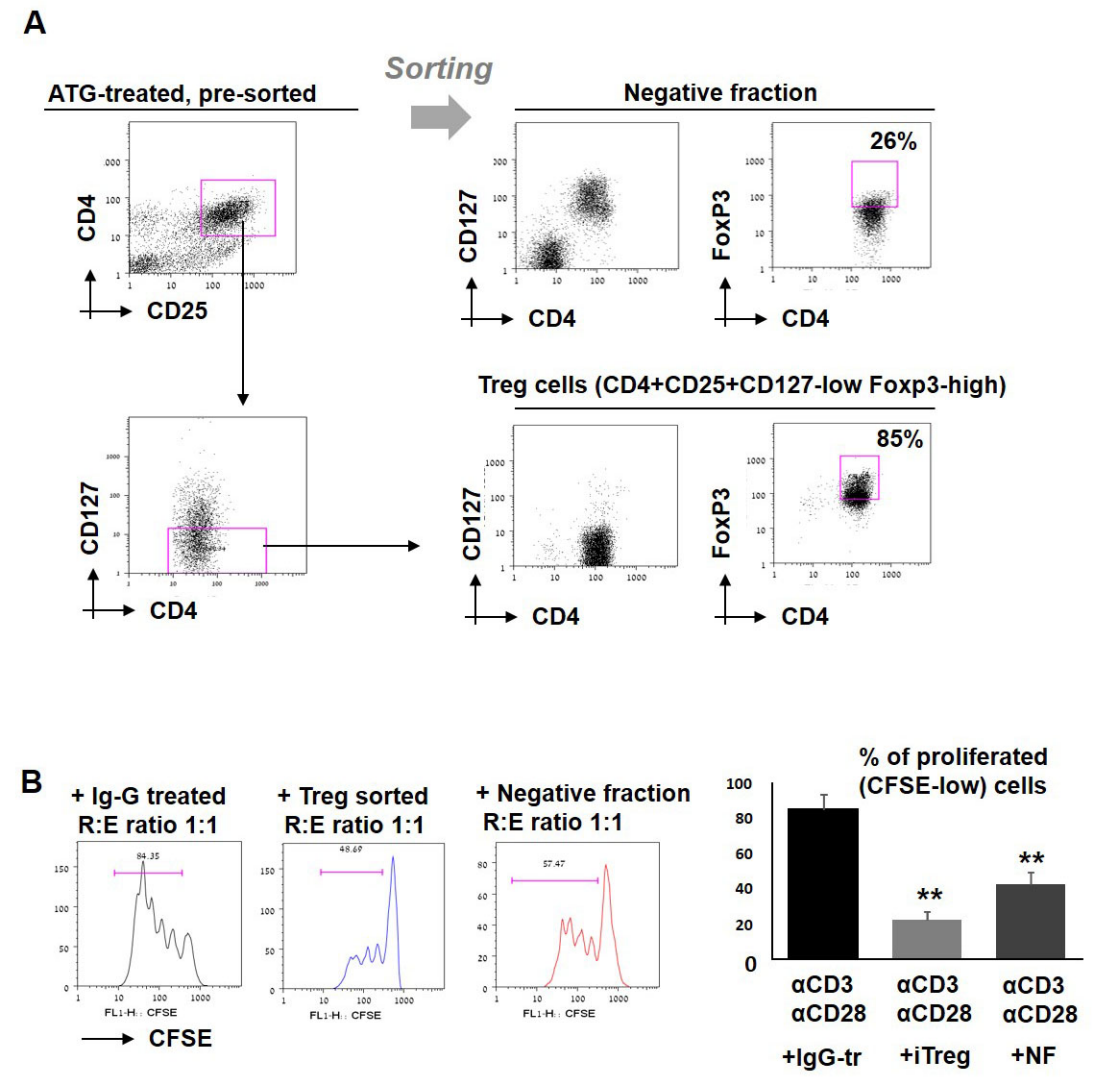
Effect of sorted iTreg cells on T cell proliferation **A.** PBMCs were incubated during 48 hours with ATG (60 µg/ml), stained for CD4, CD25 and CD127, and separated into two fractions: CD4+CD25+CD127- cells (enriched with viable Treg cells) and negative fraction. Treg phenotype was confirmed by FoxP3 staining. **B.** Sorted enriched Treg cells and negative fraction were applied to the stimulated autologous PBMCs and proliferation was measured by CFSE staining. Percentage of proliferated cells is presented by bars as mean of triplicates ±STDEV (**p < 0.01). Data are representative of two independent experiments. Tr = treated.

Subsequently, in order to enrich the tolerizing population from ATG-treated bulk culture, sorting of CD4+CD25+CD127-low cells (considered as viable regulatory T cells [42]) from ATG-treated PBMCs was performed. Treg phenotyping of the enriched fraction was further confirmed by Foxp3 staining, demonstrating 85% Foxp3 positive cells in the enriched Treg fraction and only 26% FoxP3-expressing cells in the Treg-negative fraction (Figure 6A). Enriched iTreg+ cells demonstrated a greater suppressive potency than Treg-depleted fraction when added to the autologous stimulated PBMCs (Figure 6B). Of note, Treg-depleted fraction was still able to suppress the proliferation, albeit less efficiently then sorted Treg cells, suggesting that ATG is capable to induce multiple immune suppressive cell populations. Remaining “contaminating” Treg cells may provide another explanation for the suppressive activity of the negative fraction.

### Soluble factors secreted by ATG-primed cells are involved in iTreg-mediated tolerance

**Figure 7:**
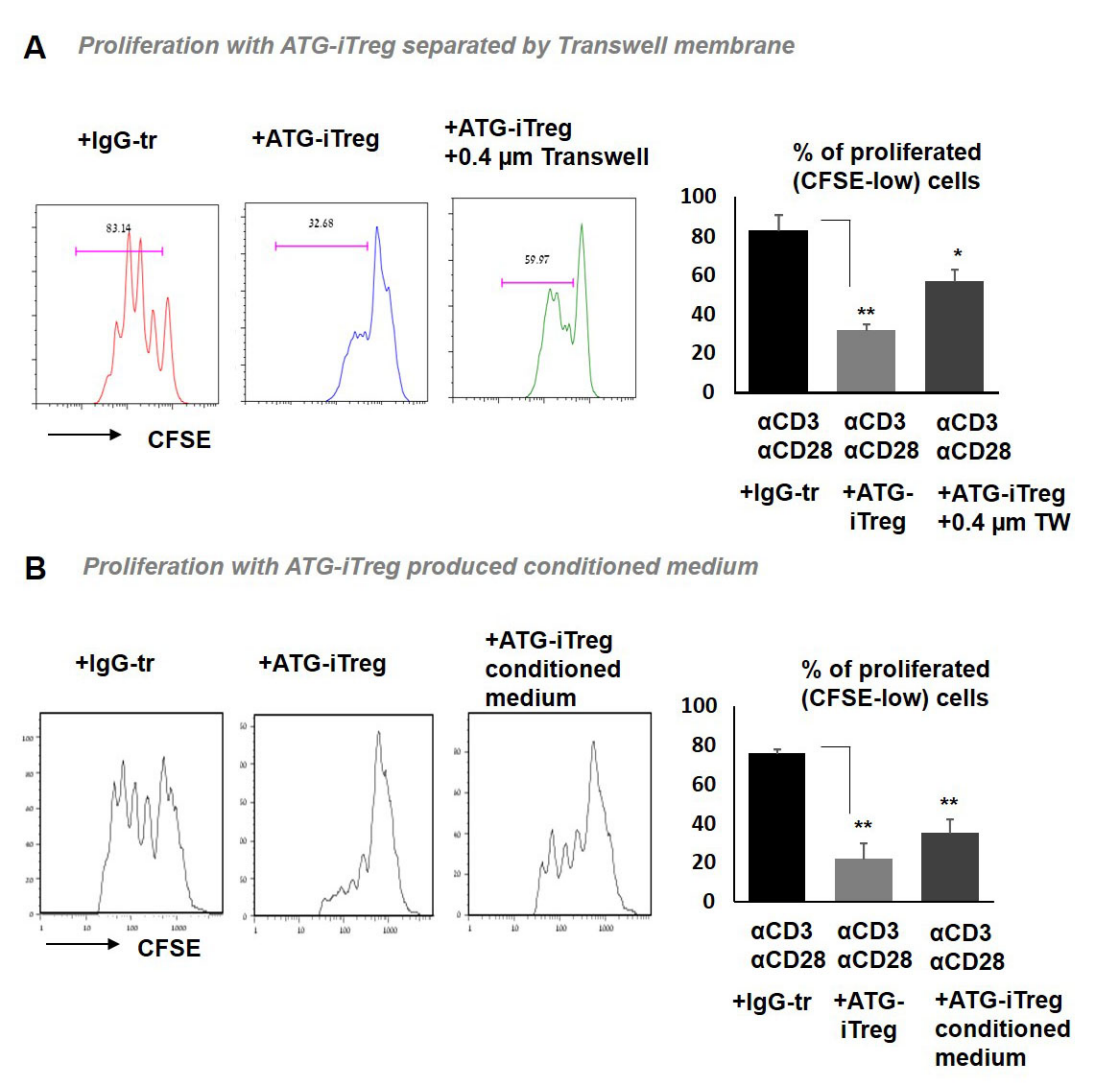
Soluble factors secreted by ATG-primed cells are involved in iTreg-mediated tolerance Proliferation of autologous PBMCs stimulated with anti-CD3/anti-CD28 antibodies in the presence of **A.** ATG-primed Treg cells, plated in direct contact or separated with 0.4 µm transwells (TW), or **B.** in the presence of iTreg-produced 50% conditioned medium. For conditioned medium generation, PBMCs were incubated during 48 hours with ATG (60 µg/ml), ATG was washed out and cell were re-plated in fresh medium for additional 48 hours. Percentage of CFSE-low proliferated cells is presented as mean of triplicates ±STDEV (**p < 0.01). Data are representative of two independent experiments. Tr = treated.

To explore the possible contribution of soluble factor(s)-mediated mechanisms, in addition to cell to cell contact mechanisms, two approaches were undertaken. First, we examined the suppressive capacity of ATG-induced Treg cells separated from the anti-CD3/anti-CD28-stimulated CFSE-labeled PBMCs by a permeable membrane. When Treg cells were placed in the top chamber of 0.4 µm transwells, their ability to suppress T cell proliferation located in the lower chamber was impaired, in comparison to the suppressive effect we observed in the direct contact standard co-culture experiments, but it was still significant in comparison to the proliferation with addition of IgG-treated control cells (Figure 7A). Similarly, conditioned medium (CM) produced by ATG-primed cells that was added to the stimulated autologous PBMCs was able to significantly suppress their proliferation, albeit less efficiently than direct contact with iTreg cells (Figure 7B). Altogether, these results suggest the presence of soluble factors secreted by ATG-primed cells that are possibly involved in the observed suppression.

### TGFβ signaling inhibition reverses the suppressive effect of ATG-induced Treg cells

**Figure 8:**
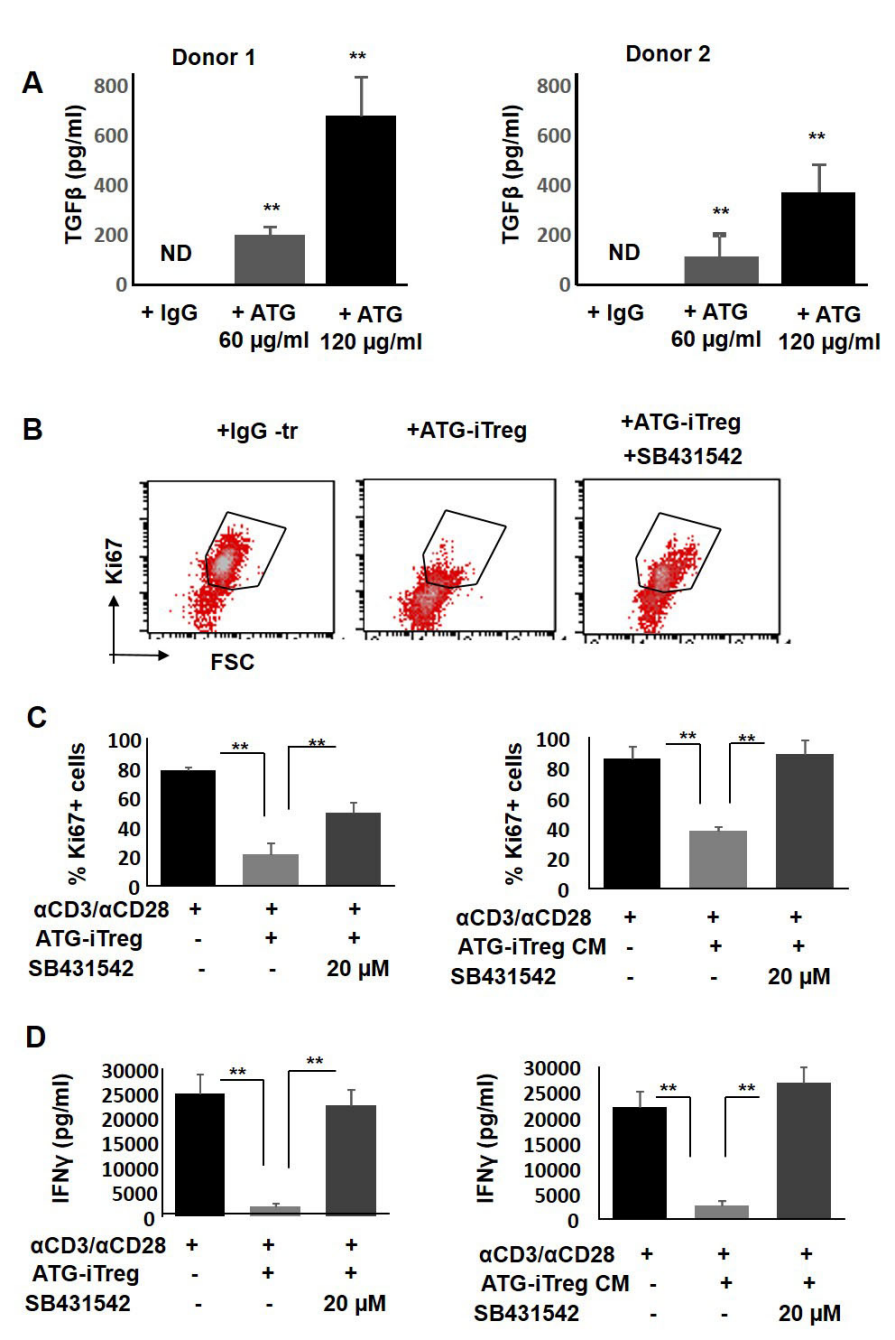
The role of TGFβ in ATG-related Treg induction and their suppressive capacity **A.** TGFβ secretion from ATG -treated PBMCs. PBMCs were treated during 48 hours with indicated concentration of ATG and TGFβ was assessed using ELISA method. **B.**-**C.** Proliferation of PBMCs in response to anti-CD3/anti-CD28 stimulation, in the absence or presence of autologous ATG-primed iTreg cells or addition of 50% conditioned medium (CM) produced by ATG-pretreated iTreg cells in the absence or presence of TGFβ receptor kinase inhibitor SB431542 (10 or 20 µM), during 5 days of culture. (B) Representative flow cytometry plots demonstrating Ki67 expression. (C) Percentage of proliferated Ki67-poistive cells is presented by bars as mean of triplicates ±STDEV (**p < 0.01). **D.** Activation measured by IFNγ levels secreted in the absence or presence of TGFβ receptor kinase inhibitor SB431542, using ELISA. Data is presented as mean of triplicates ±STDEV, **p < 0.01. Data are representative of three independent experiments.

One of the critical soluble factors known to be produced by Treg cells and being involved in immune suppression is TGFβ [43]. In order to assess the possible involvement of TGFβ signaling in the tolerance mediated by ATG-induced Treg cells, secretion of TGFβ in response to ATG treatment was evaluated. As demonstrated in Figure 8A, a significant dose-dependent increase in TGFβ levels was detected by ELISA in the culture medium of ATG-treated PBMCs. Next, in order to test the functional role of TGFβ in our assay and neutralize TGFβ signaling, we used the selective TGFβ receptor kinase inhibitor SB431542 [44]. Addition of TGFβR inhibitor to the proliferation assay was able to reverse the suppressive effect of Treg cells, thereby increasing the percent of Ki67+ proliferating T cells. Inhibition of TGFβ signaling effectively abrogated the suppression promoted by both ATG-induced Treg cells and Treg-produced CM (Figure 8B, 8C). Suppression of IFNγ secretion mediated by ATG-induced Treg cells or their CM was reversed upon the addition of SB431542 (Figure 8D). These results indicate the importance of TGFβ signaling being part of the suppressive mechanism mediated by ATG-primed Treg cells.

### TGFβ signaling and IL-2 signaling are important for ATG-mediated Treg generation

**Figure 9:**
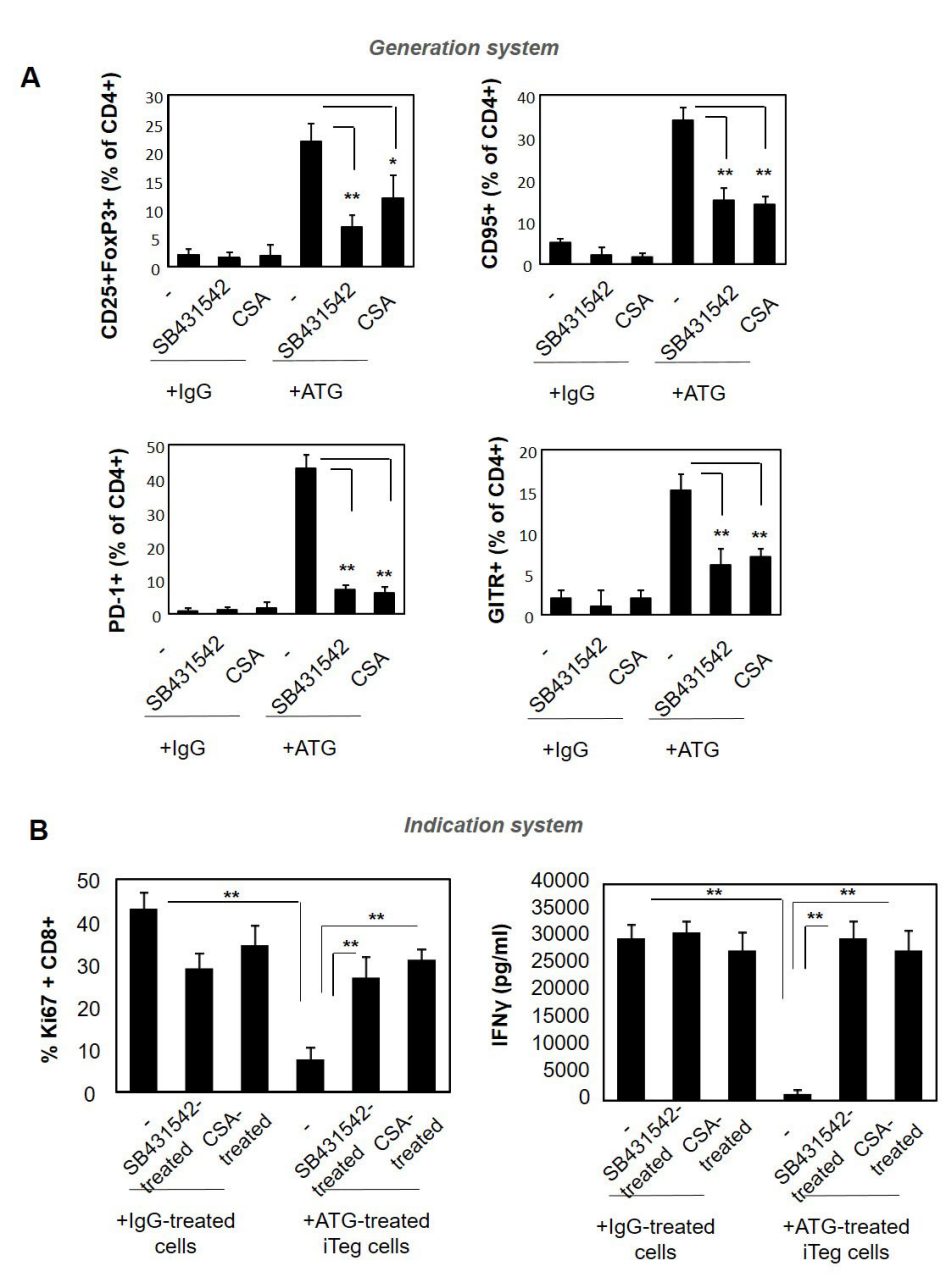
Pre-treatment with SB431542 or CSA interferes with ATG-mediated Treg induction PBMCs were incubated during 48 hours with either IgG or ATG (60 µg/ml), in the absence or presence of SB431542 (20 µM) or CSA (1 µg/ml ). **A.** Expression of Treg markers was evaluated by flow cytometry. Percentage of CD4+ cells expressing CD25+/Foxp3, CD95, PD-1 and GITR are presented as mean of triplicates ±STDEV, **p < 0.01. **B.** ATG-induced Treg cells, cultured in the absence or presence of SB431542 or CSA were applied on autologous PBMCs (1:1 ratio) and stimulated with anti-CD3/anti-CD28 for five days. Proliferation and activation were assessed by Ki67 staining and IFNγ secretion measurement, respectively. Percentage of proliferated Ki67-poistive cells is presented as mean of triplicates ±STDEV (**p < 0.01). Data are representative of three independent experiments.

It is known that TGFβ not only mediates the suppression promoted by Treg cells, but also induces the acquisition of Foxp3+ Treg phenotype [45, 46]. IL-2 is an additional factor that is important for the generation and survival of Treg cells. Therefore, our next aim was to assess the involvement of TGFβ and IL-2 signaling in the ATG-promoted acquisition of Treg phenotype. To this end, we added the TGFβ receptor kinase inhibitor SB431542 and cyclosporine A (CSA) to the Treg generation system, in combination with ATG or control IgG. Treatment with both inhibitors interfered with Treg phenotype acquisition upon ATG induction, and resulted in reduced expression levels of CD25, FoxP3, CD95, PD-1 and GITR markers (Figure 9A). Furthermore, iTregs that were pre-treated with ATG in combination with SB431542 or CSA were not able to suppress the proliferation and IFNγ secretion in response to anti-CD3/anti-CD28 stimulation (Figure 9B). These data suggest the pivotal role of the TGFβ and IL-2 signaling pathways in the acquisition of regulatory phenotype upon ATG treatment.

## DISCUSSION

Since their discovery and based on their properties, Tregs have been an attractive potential cell subset for tolerance induction in different clinical scenarios. In preclinical models of HSCT, Tregs have demonstrated their ability to prevent GVHD while preserving graft *versus* tumor effects [16, 18, 19, 47, 48]. Different approaches have been attempted for the use of Treg cells in prevention or treatment of acute and chronic GVHD, including the adoptive transfer of Treg cells from different donor sources [22, 49] or enhancement of *in vivo* Treg function by administration of low doses of IL-2 [50].

The role of ATG in Treg induction has been previously described with recent publications, as well as our own work, indicating that exposure of lymphocytes to ATG not only causes depletion of effector T cells but also induces the preferential expansion of human CD4+CD25+Foxp3+ Tregs *in vitro* [35-38]. On the other hand, a study by Broady and co-workers did not provide evidence for the existence of thymoglobulin-induced Treg cells but rather related the observed increase in FoxP3 expression to an activated phenotype [39]. Challenging this discriminatory data, here we clearly demonstrate that *in vitro* treatment of PBMCs with ATG-F is capable of increasing the frequency of functional Treg cells, which are able to suppress the proliferation and activation of autologous and allogeneic stimulated T cells.

Importantly, we found that ATG treatment significantly increases the surface expression of complement regulatory molecules CD55, CD58 and CD59 on the generated Treg cells. Previous works show that in contrast to effector T cells, complement components rather activate and expand Treg cells [51]. CD55 was identified as co-stimulator and activator of human naive CD4+ cells, resulting in the differentiation of a discrete Treg population [52]. CD58 co-stimulation (together with was CD2) was found to be important for Treg function [53]. Therefore, the observed up-regulation of CD55, CD58 and CD59 expression on ATG-induced Treg cells potentially can lead to increased resistance of Treg to complement-mediated lysis, and conversely to increased FoxP3 expression and Treg function.

Little is known about the mechanisms underlying the effects promoted by ATG to induce Treg as well as the mechanism underlying tolerogenic activity of these cells. Therefore, our work was directed to decipher the mechanism of action of ATG-induced Treg cells. Several mechanisms have been proposed for the observed Treg-induced tolerance and the suppressive mediators. Production of suppressive cytokines, including IL-10 and TGFβ have been shown to be important in Treg-mediated suppression [54, 55]. Treg function can be also mediated by cell to cell contacts, utilizing contact-dependent molecules, such as CTLA-4, granzymes and perforins [56, 57]. However, the mechanism by which ATG-induced Treg cells promote tolerance and suppress effector T cell activation and proliferation has not been fully elucidated yet. Here we demonstrate the importance of TGFβ signaling in both phases: the initial Treg phenotype acquisition promoted by ATG and the subsequent suppression mediated by induced Treg cells were both interfered upon TGFβ inhibition. TGFβ blockade with a specific TGFβ receptor kinase inhibitor was able to prevent the effect of ATG and resulted in reduced expression of Treg markers in ATG-treated cells. Similarly, inhibition of TGFβ with SB431542 impaired the immune suppression promoted by ATG-induced Treg cells and reversed the proliferation and activation of autologous T cells in response to anti-CD3/CD28 stimulation. Previous studies that assessed the possible involvement of TGFβ in ATG-related Treg expansion demonstrated some controversy regarding the role of TGFβ. In one study using thymoglobulin no TGFβ gene expression was observed in PBMCs upon treatment with theanti-thymocyte globulin [58]. Another study evaluated the effect of thymoglobulin on cytokine secretion in *ex vivo* treated T cells, and detected mainly IL-10 increase without TGFβ secretion in response to the treatment [59]. In contrast, increase in TGFβ expression following ATG treatment was reported in several other studies. For instance, clinical study in renal allograft recipients treated with ATG-F demonstrated increase in TGFβ gene expression in PBMCs [60]. Different sources of ATG and different experimental protocols may explain the discrepancies. Nevertheless, our findings support the importance of TGFβ pathway in ATG-mediated tolerance and suggest its possible involvement in clinical settings of allogeneic HSCT. Complementing the results of previous studies we now show the mechanistical link between ATG-induced Treg generation, the release of TGFβ and its immunosuppressive activity.

ATG is polyclonal and can recognize multiple antigens and affect different cell populations. Identification and isolation of specific defined and potent immunosuppressive populations is important step toward better understanding of ATG-related properties and efficacious application in the clinical settings. We show here that viable CD4+CD25+CD127-low Treg cells isolated from bulk culture of ATG-treated PBMCs demonstrate preferential suppressive activity comparing to non-sorted population. However, it was interesting to learn that residual negative fraction of ATG-treated cells is also able to suppress the proliferation of autologous cells, albeit less efficient than purified Treg cells. These results suggest that in addition to Treg cells, ATG may have an impact on other suppressive populations. Indeed, it was demonstrated that ATG-mediated Treg expansion requires the presence of monocytes in culture, and generation of tolerogenic CD14+CD11c+ dendritic cells was detected upon ATG treatment [58]. The role of dendritic cells in the induction of lymphocyte tolerance is established phenomenon [61]. However, the role of ATG and its effects on different immune cell populations, including tolerogenic myeloid cells, should be further characterized and possibly utilized in anti-GVHD therapeutics development.

Numerous factors may affect a given patient’s response to ATG treatment. The type of the pre-transplantation conditioning, the type of GHVD prophylaxis, the number and type of immune cells in the patient’s peripheral blood and the immune cell composition of the graft - all these factors may significantly impact the ATG-mediated effects and clinical responses and future research needs to address these issues.

Among immunosuppressive agents, it was shown that CSA has a negative impact on Treg cells, impairing their inhibitory activity [36, 62]. CSA, which disturbs IL-2 signaling, but not rapamycin, has been shown to suppress the ability of Treg cells to inhibit the proliferation of stimulated PBMCs [63]. Consistently, in our experiments, the combination of ATG with CSA was found to be unfavorable for Treg induction. To this end, CSA treatment interfered with ATG-promoted effects, significantly reducing the induction of Treg markers upon ATG treatment, and suppressing the inhibitory activity Treg cells.

Recent work had demonstrated the importance of ATG binding to immune cell populations following allogeneic HSCT. It has been shown that high levels of ATG antibodies bound to T and B cells on day 7 post-transplantation are associated with a low risk of aGVHD [64]. Among all immune cells, ATG has the highest affinity for naïve T cells [65], which are enriched for alloreactive T cells [66]. However, in addition to depletion of effector T cells transferred with graft, ATG binding may result in Treg induction, possibly affecting either donor as well as recipient T cells. Our recent *in vitro* results demonstrate the suppressive potential of ATG-generated Treg against either autologous or allogeneic cells. We suggest that ATG-induced regulatory T cells of both patient and graft origin may suppress the activation and proliferation of alloreactive donor T cells, although we do not have yet formal prove for this. Another question to be explored is the influence of Treg in general and ATG-induced Treg, more specifically, on graft *versus* tumor (GVT) effect.

Altogether, we demonstrate here that ex vivo treatment with ATG potently induces expansion of functional Treg cells which are able to suppress the proliferation and activation of both autologous and allogeneic effector T cells. We found that ATG-mediated Treg induction and activity was partially dependent on TGFβ signaling. Therefore, the therapeutic effects of ATG in the settings of allogeneic HSCT may be the result of not only lymphocyte depletion but also enhanced Treg cell number and function, and may be important for GVHD prevention and efficient engraftment.

Further insight into the basic biology of Treg cells will aid in translating preclinical data for the benefit of patients.

## MATERIALS AND METHODS

### Immunosuppressive drugs and control antibodies

Following drugs and inhibitors were used in the study: rATG (Neovii-Biotech), a purified IgG fraction derived from rabbits immunized with Jurkat T cell line. Control rabbit IgG purified from whole normal rabbit serum was used (Sigma-Aldrich, Israel). Cyclosporine A was purchased from Sigma-Aldrich, SB431542 from Cayman.

### Cell isolation and induction culture

Heparinized blood samples from healthy volunteers were collected after written informed consent. The the study was approved by the institutional review board and Israeli Ministry of Health. Peripheral blood mononuclear cells (PBMCs) were isolated by standard Ficoll density gradient centrifugation. For Treg induction, PBMCs (10^6^/mL) were incubated with rATG, or rIgG, (each at 60 μg/mL or 120 μg/mL), in the presence or absence of CSA (1.0 μg/mL) or SB431542 in RPMI 1640 medium supplemented with 10% heat-inactivated fetal calf serum at 37°C, 95% humidity, and 5% CO_2_ for 48 hours. Supernatants were collected for cytokine detection. Cells were collected for flow cytometry analysis or functional assays.

### Flow cytometry analysis

Expression of markers associated with Treg phenotype was evaluated by flow cytometry. Briefly, PBMCs were first stained with antihuman CD4-fluorescein isothiocyanate (FITC) and CD25-phycoerythrin (PE) (eBioscience, San Diego, CA). As a next step, intracellular staining for FOXP3 detection was performed using antihuman FOXP3-allophycocyanin (APC; PCH101FOXP3) commercial kit according to manufacturer’s instructions (eBioscience). Additional markers were analyzed, including CD3-FITC, CD8-PE-CY7, CD69-PE, CD95-FITC, GITR-PE, ICOS-PE-CY7, PD-1-FITC, CD55-APC, CD58-APC, CD59-APC (purchased from eBioscience, Biolegnd or TONBO). Finally, cells were analyzed on the FACSCalibur (Becton Dickinson) using the CellQuest software (BD Biosciences) and FlowJo software.

### Isolation of viable Treg cells

Following the induction treatment with ATG, cells were co-stained with anti-CD4-FITC, anti-CD25-PE and anti-CD127-APC and sterile sorting of CD4+CD25+CD127-low cell population (considered as positive fraction enriched with viable Treg cells, [42] versus mixed population of CD4+CD127-high cells together with CD4-negative cells (considered as negative fraction) was performed using FACSAria II cell sorter (Becton Dickinson). Cell purity was confirmed using subsequent Foxp3 of sorted cell populations. Sorted cells were proceeded for functional assays.

### Proliferation assays

To assess the suppressive capacity of unsorted and sorted ATG-induced Treg cells, the cells were co-cultured with autologous or allogeneic PBMCs (responders) at 1:1 ratio in the presence of 1 μg/mL soluble anti-CD3 (OKT3, eBioscience) and 1 μg/mL soluble anti-CD28 (CD28.2, eBioscience). After culture for five days, proliferation was evaluated using Carboxyfluorescein diacetate succinimidyl ester (CFSE) dilution method or by Ki67 intra-cellular staining. For CFSE staining, prior to the incubation cells were labeled with 5 μM CFSE (eBioscience) according to manufacturer’s instructions and analyzed five days later by flow cytometry for the loss of fluorescence. For Ki67 detection, cells were harvested following the proliferation and subjected to intra-cellular staining using FOXP3 fixation and permeabilization buffers kit (eBioscience) and anti-Ki67-APC conjugated antibody (eBioscience).

### Conditioned medium generation and experiments with transwells

To evaluate the effect of secreted factors produced by iTreg cells, conditioned medium (CM) was generated. PBMCs (10^6^/mL) were pre-treated with either IgG or ATG for 48 hours, washed three times with PBSx1 and re-plated in fresh medium for additional 48 hours. Culture supernatants were isolated by centrifugation to remove cellular contamination prior to use in assays. Transwell experiments were performed using 0.4 µm pore size Transwell™ system (Corning, Costar). Responder PBMCs were plated in the down chamber, IgG- or ATG-pretreated iTreg cells were placed in the upper chamber. Cells were stimulated with anti-CD3/anti-CD28 for 5 days and proliferation was assessed as described above.

### Enzyme-linked immunosorbent assay (ELISA)

The levels of INFγ or TGFβ in the supernatants of treated cells were measured using commercially available ELISA kits according to the manufacturer’s instructions (eBioscience). Samples and standards were tested in triplicates.

### Statistical analyses

Data are expressed as the mean ± standard deviation (STDEV). Statistical comparisons of means were performed by a two-tailed unpaired Student’s *t* test.
